# Amivantamab Compared with Real-World Physician’s Choice after Platinum-Based Therapy from a Pan-European Chart Review of Patients with Lung Cancer and Activating *EGFR* Exon 20 Insertion Mutations

**DOI:** 10.3390/cancers15225326

**Published:** 2023-11-08

**Authors:** Petros Christopoulos, Nicolas Girard, Claudia Proto, Marta Soares, Pilar Garrido Lopez, Anthonie J. van der Wekken, Sanjay Popat, Joris Diels, Claudio A. Schioppa, Jan Sermon, Nora Rahhali, Corinna Pick-Lauer, Agnieszka Adamczyk, James Penton, Marie Wislez

**Affiliations:** 1 Thoraxklinik and National Center for Tumor Diseases at Heidelberg University Hospital, 69126 Heidelberg, Germany; 2 German Center for Lung Research (DZL), 35392 Gießen, Germany; 3 Institut Curie, Institut du Thorax Curie-Montsouris, 75005 Paris, France; nicolas.girard2@curie.fr; 4 Paris Saclay University, University of Versailles Saint-Quentin-en-Yvelines (UVSQ), 78000 Versailles, France; 5 Medical Oncology Department, Fondazione IRCCS Istituto Nazionale dei Tumori, 20133 Milan, Italy; claudia.proto@istitutotumori.mi.it; 6 Instituto Português de Oncologia do Porto Francisco Gentil, 4200-072 Porto, Portugal; martasoares@ipoporto.min-saude.pt; 7 Instituto Ramón y Cajal de Investigación Sanitaria (IRYCIS), Hospital Universitario Ramón y Cajal, 28034 Madrid, Spain; pilargarridol@gmail.com; 8 University of Groningen, University Medical Centre Groningen, 9713 GZ Groningen, The Netherlands; a.j.van.der.wekken@umcg.nl; 9 The Royal Marsden Hospital, London SW3 6JJ, UK; sanjay.popat@rmh.nhs.uk; 10 The Institute of Cancer Research, London SW7 3RP, UK; 11 Janssen Pharmaceutica NV, 2340 Beerse, Belgium; jdiels@its.jnj.com (J.D.); cschiopp@its.jnj.com (C.A.S.); jsermon@its.jnj.com (J.S.); 12 Janssen-Cilag Ltd., 92130 Issy-les-Moulineaux, France; nrahhali@its.jnj.com; 13 Janssen-Cilag GmbH, 41470 Neuss, Germany; cpick@its.jnj.com; 14 Janssen-Cilag Ltd., 28042 Madrid, Spain; aadamczy@its.jnj.com; 15 Janssen-Cilag Ltd., High Wycombe HP12 4EG, UK; jpenton1@its.jnj.com; 16 Hôpital Cochin, APHP, 75014 Paris, France; marie.wislez@aphp.fr

**Keywords:** adjusted comparison, amivantamab, *EGFR* Exon 20 insertion mutations, non-small cell lung cancer, real-world physician’s choice

## Abstract

**Simple Summary:**

Patients with advanced non-small cell lung cancer (NSCLC) with epidermal growth factor receptor gene (*EGFR*) Exon 20 insertion mutations (Exon20ins) used to have poor outcomes after the failure of platinum-based therapies. Amivantamab, a drug that targets two proteins involved in NSCLC (EGFR/MET), was developed and studied in the CHRYSALIS clinical trial. This paper summarizes an analysis comparing outcomes for amivantamab from CHRYSALIS to the outcomes for a mix of treatments that were used in real-world clinical practice (real-world physician’s choice [RWPC]). RWPC data were collected from hospitals across Europe in the CATERPILLAR real-world evidence (RWE) study. The outcomes of 114 patients from CHRYSALIS were compared to 55 lines of treatment (LOTs) from CATERPILLAR-RWE, accounting for differences in patient characteristics between the two populations. Overall, the results show that amivantamab was at least twice as good as RWPC for platinum pre-treated patients in terms of tumor response and survival rates.

**Abstract:**

Patients with advanced non-small cell lung cancer (NSCLC) with epidermal growth factor receptor gene (*EGFR*) Exon 20 insertions (Exon20ins) at the second line and beyond (2L+) have an unmet need for new treatment. Amivantamab, a bispecific EGFR- and MET-targeted antibody, demonstrated efficacy in this setting in the phase 1b, open-label CHRYSALIS trial (NCT02609776). The primary objective was to compare the efficacy of amivantamab to the choices made by real-world physicians (RWPC) using an external control cohort from the real-world evidence (RWE) chart review study, CATERPILLAR-RWE. Adjustment was conducted to address differences in prognostic variables between cohorts using inverse probability weighting (IPW) and covariate adjustments based on multivariable regression. In total, 114 patients from CHRYSALIS were compared for 55 lines of therapy from CATERPILLAR-RWE. Baseline characteristics were comparable between the amivantamab and IPW-weighted RWPC cohorts. For amivantamab versus RWPC using IPW adjustment, the response rate ratio for the overall response was 2.14 (*p* = 0.0181), and the progression-free survival (PFS), time-to-next-treatment (TTNT) and overall survival (OS) hazard ratios (HRs) were 0.42 (*p* < 0.0001), 0.47 (*p* = 0.0063) and 0.48 (*p* = 0.0207), respectively. These analyses provide evidence of clinical and statistical benefits across multiple outcomes and adjustment methods, of amivantamab in platinum pre-treated patients with advanced NSCLC harboring *EGFR* Exon20ins. These results confirm earlier comparisons versus pooled national registry data.

## 1. Introduction

Lung cancer is the leading cause of cancer-related mortality and one of the most prevalent cancers worldwide [1]. Non-small cell lung cancer (NSCLC) accounts for approximately 85% of all lung cancer cases [2]. An estimated 15% of Western European patients with NSCLC harbor mutations in the epidermal growth factor receptor gene (*EGFR*) [3]. Common *EGFR* mutations (*EGFR+*), including Exon 19 deletions and p.L858R, occur in approximately 80–85% of *EGFR+* cases; Exon 20 insertions (Exon20ins) and other rare mutations account for the remaining ~15–20% [4]. Current estimates of the prevalence for Exon20ins are between 4 and 10% of *EGFR*+ NSCLC; this is contingent on genotyping technology [3,5,6].

Patients with Exon20ins are often described as having similar clinical characteristics to other *EGFR+* patients in real-world analyses [6]. However, their prognosis is worse as they may have poor responses to conventional EGFR-tyrosine kinase inhibitors (TKIs) and, thus, are in need of new treatment options [6,7,8,9]. A post hoc analysis of the LUX-Lung study series demonstrated that Exon20ins patients receiving afatinib had reduced median progression-free survival (PFS) when compared with patients with common *EGFR* mutations (2.7 months [95% confidence interval [CI] 1.8, 4.2] versus 10.7 months [95% CI 5.6, 14.7]) [10]. Osimertinib is also used in clinical practice, but has demonstrated limited clinical activity, with an overall response rate of ~25% or less in patients with advanced NSCLC and *EGFR* Exon20ins [11,12,13].

Until recently, there has been a lack of effective treatment options for advanced NSCLC patients with *EGFR* Exon20ins [2]. For second line or later (2L+), after the receipt of platinum-based therapy, physicians in Europe used conventional EGFR TKIs, immuno-oncology (IO) agents, or various chemotherapies, including retreatment with platinum-based regimens [13,14]. This heterogenous mix of treatments is hereafter referred to as real-world physician’s choice (RWPC). In recent years, two targeted therapies (amivantamab and mobocertinib) have become available for the treatment of these patients [15,16].

Amivantamab is a bispecific EGFR- and MET-targeted antibody. The CHRYSALIS trial with a phase 1b, single arm, open-label, multicenter, multicohort trial (NCT02609776) demonstrated the safety and efficacy of amivantamab for treating patients with locally advanced or metastatic NSCLC with *EGFR* Exon20ins mutations, including patients for whom platinum-based chemotherapy had previously failed (Cohort D+) [15,17]. Based on the results from Cohort D+ for CHRYSALIS, regulatory approval was granted in this setting by the US Food and Drug Administration (FDA), the European Medicines Agency (EMA), the UK Medicines, and Healthcare products Regulatory Agency (MHRA) in 2021 and other regulatory bodies [15,18,19]. The efficacy and safety of mobocertinib, an oral and irreversible EGFR-TKI, as a treatment for locally advanced or metastatic NSCLC with *EGFR* Exon20ins mutations in patients who progressed on platinum-based chemotherapy was assessed in the phase 1/2, single-arm, open-label, multicenter EXCLAIM (NCT02716116) study [20]. Mobocertinib received approval by the FDA (through accelerated approval) and conditional approval by the MHRA [16,21]. However, mobocertinib is being voluntarily withdrawn in the US due to failure to meet its primary endpoint in the phase 3 EXCLAIM-2 trial (NCT04129502) [22,23]. In addition, mobocertinib is not approved by the EMA, following a withdrawal of the application for conditional marketing authorization by the manufacturer [24].

Following the development of these targeted drugs, both amivantamab and mobocertinib are recommended within relevant treatment guidelines as monotherapy for patients with *EGFR* Exon20ins-mutated NSCLC who have progressed on or after platinum chemotherapy. For example, the European Society for Medical Oncology (ESMO) Clinical Practice Guideline for the treatment of oncogene-addicted metastatic NSCLC states that amivantamab is recommended as a treatment (IIIB, Magnitude of Clinical Benefit [MCBS] 3, Scale for Clinical Actionability of molecular Targets [ESCAT] IB) and mobocertinib can be given as a treatment (IIIC, MCBS 2, ESCAT IB) for *EGFR* Exon20ins-mutated NSCLC after the failure of prior platinum-based therapy, noting that mobocertinib is not approved by the EMA [25].

Given the rarity of tumors with *EGFR* Exon20ins mutations and the limitations associated with identifying these mutations via conventional polymerase chain reaction-based methods, recruiting large patient cohorts in this setting for a randomized trial is challenging [2,26]. Moreover, competitive recruitment for clinical studies in this patient population makes a confirmatory randomized trial unfeasible for these patients after the failure of platinum chemotherapy. In addition, there are difficulties associated with identifying an appropriate comparator arm for a randomized trial due to the prior lack of a clear standard of care in this setting. As such, this necessitates the use of a real-world external cohort to determine the relative efficacy of amivantamab compared to RWPC [27]. Real-world evidence (RWE) analyses (such as retrospective chart reviews and prospective external cohorts) have been utilized in the support of regulatory approval and HTA submissions of other oncology therapies, demonstrating their usefulness [27,28,29].

Efficacy data comparing amivantamab versus RWPC have previously been generated based on both US-based real-world sources and national registry data from seven European countries and US real-world sources [14,30]. To complement these comparisons versus national registry data, a pan-European, multicenter chart review study was conducted in patients with advanced NSCLC and *EGFR* Exon20ins mutations following platinum-based therapy to enable an adjusted treatment comparison of amivantamab versus RWPC in Europe. This publication reports the results of this comparison and demonstrates the clinical and statistical benefit of amivantamab compared to RWPC in patients with advanced NSCLC and *EGFR* Exon20ins at 2L+.

## 2. Materials and Methods

The primary objective of this analysis was to compare the efficacy of amivantamab in patients with advanced *EGFR* Exon20ins-mutated NSCLC following platinum-based therapy at 2L+, as assessed in Cohort D+ of the CHRYSALIS study, to RWPC, which was assessed using an external control cohort of patients identified in the RWE chart review study CATERPILLAR-RWE.

### 2.1. Amivantamab Cohort

For this analysis, the amivantamab cohort included patients with NSCLC with an *EGFR* Exon20ins mutation, who had an Eastern Cooperative Oncology Group (ECOG) performance status of 0 or 1 and who progressed on or after previously receiving platinum-based chemotherapy, and received their first dose of amivantamab monotherapy either on or before 04 June 2020 at the recommended phase 2 monotherapy dose regimen (as per Cohort D+ of the trial [1050 mg for body weight < 80 kg and 1400 mg for body weight ≥ 80 kg]). Patients who received prior immunotherapy as part of chemotherapy in combination with an IO were included. Key eligibility criteria for CHRYSALIS Cohort D+ are presented in Table S1. For patients in the amivantamab cohort, the index date was the date of the first amivantamab dose administration.

CHRYSALIS data used in the analyses were from the 30 March 2021 data cut for overall response rate (ORR), PFS and time-to-next treatment (TTNT) and the 7 March 2022 data cut for overall survival (OS). These represent the most recent data available at the time of analysis. The median duration of follow-up was 12.5 months and 24.9 months for the 30 March 2021 data cut and the 7 March 2022 data cut, respectively. Given the similarity in results assessed by the investigator assessment (INV) and blinded independent review committee (IRC) for both ORR and PFS from CHRYSALIS, analyses comparing INV only between amivantamab and RWCP are presented within this manuscript.

### 2.2. CATERPILLAR-RWE Cohort

CATERPILLAR-RWE is a pan-European, non-interventional, multicenter chart review study designed to provide an external control cohort for the CHRYSALIS trial.

Data were derived from existing medical records to characterize the patterns and effectiveness of real-world treatment approaches among patients in Europe similar to those enrolled in the CHRYSALIS trial. This study was implemented in 22 sites across Spain (six sites), the UK (five), Italy (four), France (three), Germany (two), Portugal (one), and The Netherlands (one) for patients who had been treated during the period 1 January 2011 to 31 May 2021. The final analysis set included 17 sites across Spain (four sites), the UK (three), Italy (three), France (three), Germany (two), Portugal (one) and The Netherlands (one).

The CATERPILLAR-RWE cohort was identified from multiple sources including electronic medical record (EMR) data and/or medical records available in other formats within hospitals, such as paper records, laboratory results, scans, or other imagery. Data were abstracted from EMR data onto an electronic case report form; case report forms were used to collect the data, rather than a direct link to EMR data. Medical records from adult patients with a confirmed diagnosis of locally advanced or metastatic NSCLC between 1 January 2011 and 31 July 2020 were assessed according to the main CHRYSALIS eligibility criteria contained in Table S2, which were designed to be similar to Cohort D+ of the CHRYSALIS trial (Table S1).

The RWPC cohort contained all LOTs for patients who satisfied the eligibility criteria at initiation for each LOT. LOTs containing amivantamab and all subsequent LOTs after amivantamab were received and excluded from the analysis set.

It was not feasible to apply all of the eligibility criteria of Cohort D+ of the CHRYSALIS trial to the RWPC cohort due to the unavailability of relevant datapoints. The inclusion and exclusion criteria of Cohort D+ of the CHRYSALIS trial that could not be applied to the RWPC cohort are presented in Table S3.

Data were collected retrospectively from the diagnosis of locally advanced/metastatic NSCLC until death due to any cause, loss to follow-up, or the end of the study period (whichever occurred first). Data collected included demographic and disease characteristics, medical history, and comorbidities at the time of treatment initiation, as well as clinical outcomes.

For the comparative effectiveness analyses, the index date for the RWPC cohort was defined as the start of any LOT for which inclusion and exclusion criteria were met upon the initiation of treatment. Patients who met the selection criteria at the initiation of multiple LOTs could be included in the analysis multiple times. Including all eligible LOTs for each patient was considered the most efficient use of the available information, whereas limiting the analysis to only one line per patient, such as only the last LOT, could induce selection bias [31,32,33,34]. Only LOTs where *EGFR* Exon20ins mutation testing was performed prior to therapy initiation were retained for analysis, reflecting the treatments chosen by physicians specifically for those with *EGFR* Exon20ins mutations.

### 2.3. Endpoint Definitions

The efficacy outcomes evaluated in the analysis were ORR, PFS, TTNT and OS.

ORR was defined as the proportion of LOTs who achieved at least a partial response. PFS was defined as the interval between the index date and the date of disease progression or death from any cause, with patients censored at the last assessment before subsequent therapy if no event was observed. TTNT was defined as the time from the index date until the earliest event of initiation with the next systemic anti-cancer therapy or death, with censoring occurring at the date of the last follow-up. OS was defined as the time between the index date and date of death due to any cause and if alive, OS was censored at the date the patient was last known to be alive.

In CHRYSALIS, the primary definitions for response and progression endpoints were based on the Response Criteria in Solid Tumours (RECIST) v1.1. ORR and PFS in CHRYSALIS were both INV and IRC-assessed. Comparative analyses between CHRYSALIS and CATERPILLAR-RWE were based on INV to align with real-world clinical practice.

### 2.4. Adjustment for Differences between Patient Cohorts

To account for differences in the baseline characteristics between CHRYSALIS and CATERPILLAR-RWE patients, adjustment was carried out for key prognostic variables. Prognostic patient and disease characteristics were identified via an evidence-based process based on a systematic review of the literature and expert opinion. The covariates that were considered clinically important prognostic factors and could be adjusted for based on data availability as follows: ECOG performance status, the number of previous LOTs, bone, liver, brain, lymph node, adrenal gland, or pleural metastases, other metastatic locations, and age category (Table S4). All variables were measured at the start of LOT. Some clinically relevant covariates were not adjusted for, including the *EGFR* variant and *TP53* co-mutation; *EGFR* variant data were not systematically collected in CATERPILLAR-RWE, whilst *TP53* co-mutation data were not available in CHRYSALIS and had a high number of missing values in CATERPILLAR-RWE. Baseline variables that were not included in the adjustment are presented in Table S5.

The differences in patient and disease characteristics between cohorts were adjusted for using inverse probability weighting (IPW), specifically including the average treatment effect among the treated (ATT) approach, as well as a covariate adjustment based on multivariable regression. The imbalances before and after ATT adjustment were assessed using the standardized mean difference (SMD).

To account for the correlation of outcomes between treatment lines for patients with multiple LOTs, standard errors were calculated using the robust variance estimator [31,33,34].

#### 2.4.1. Inverse Probability Weighting

For primary effectiveness analyses, IPW was used to adjust for imbalances in prognostic patient characteristics between the amivantamab cohort and RWPC cohort. Propensity scores (PS) were estimated using a multivariable logistic regression model. These values represent the conditional probability for each patient to belong to the RWPC cohort, conditional on the baseline characteristics, and were transformed into patient-specific ATT weights.

The ATT approach was considered the most relevant as amivantamab was the intervention of relevance for the analyses. This approach allowed the estimation of (“counterfactual”) outcomes for RWPC in a patient cohort with similar baseline characteristics to the CHRYSALIS Cohort D+ (i.e., representing outcomes for CATERPILLAR-RWE if this cohort was similar to the CHRYSALIS population) [35]. ATT adjustment kept the CHRYSALIS Cohort D+ population as observed (the ‘target population’) and reweighted the CATERPILLAR-RWE cohort to match the target population. This meant that amivantamab patients received a weight of 1, while the RWPC patients were reweighted by PS/(1-PS). To maintain the original sample size for the adjusted population and reflect the associated uncertainty, ATT weights were multiplied by the ratio of the original sample size versus the sum of the ATT weights, making the sum of these recalculated weights equal to the original sample size.

For binary endpoints, the relative treatment effect in terms of the odds ratio (OR) for amivantamab versus RWPC was estimated using an ATT-weighted logistic regression model. To estimate the treatment effect in terms of the response rate ratio, a generalized linear model was used. The OR and response rate ratio a the 95% confidence interval (CI) was reported.

For time-to-event endpoints (PFS and TTNT and OS), survival probabilities were estimated using the ATT-weighted Kaplan−Meier (KM) method, and median survival times with 95% CI were reported [36]. Weighted KM graphs are presented. Hazard ratios (HR) (including 95% CI and corresponding *p*-values) for amivantamab versus RWPC were estimated using a weighted Cox proportional hazards model.

#### 2.4.2. Multivariable Regression

Covariate adjustment based on multivariable regression was used as an alternative approach to adjust for imbalances at the baseline between cohorts and potentially related confounding, with the aim of assessing consistency in results across adjustment methodologies. Moreover, a multivariable model including the same baseline characteristics adjusted for in the ATT approach as covariates, provided estimates of the prognostic value for each of these characteristics for the outcomes.

For binary endpoints, estimates for the relative treatment effect in terms of OR for amivantamab versus RWPC were estimated using a logistic regression model including prognostic variables (described in Table S4) as covariates. To estimate the treatment effect in terms of the response rate ratio, a generalized linear model was used. The OR and response rate ratio with a 95% CI were reported.

For time-to-event endpoints, HRs (including 95% CI and corresponding *p*-values) were estimated using a Cox proportional hazards model, including prognostic variables as covariates.

### 2.5. Imputation Method

For RWPC, nine (16.4%) LOTs from eight unique patients did not have information on metastatic locations.

To limit a reduction in the sample size, information on metastatic locations was imputed based on the available information from prior and subsequent LOTs (relative to the LOT with missing information) for each patient, making efficient use of all available information for each patient. The following rules were applied as follows:

- If information on metastatic locations was available from both the previous and subsequent LOT (as was the case for three LOTs) and if there was no change in the presence/absence of metastases between prior and subsequent LOT, then the available information was imputed for each metastatic location. If a change in metastatic locations between prior and subsequent LOT was present (e.g., the prior LOT did not have a metastatic location and the subsequent LOT did have a metastatic location), then the presence of metastasis was imputed.
- If information only from the prior LOT was available (four LOTs), then the last observation was carried forward.
- If information only from the subsequent LOT was available (two LOTs), then its values were imputed.

The baseline characteristics of the unadjusted (with and without imputation) RWCP cohort are presented in Table 1.

## 3. Results

### 3.1. Baseline Patient and Disease Characteristics

In total, 114 patients from CHRYSALIS were compared to 55 LOTs from 38 individual patients from CATERPILLAR-RWE (Table S6). The baseline characteristics of CHRYSALIS patients versus the unadjusted (with and without imputation) and the IPW–ATT adjusted RWPC cohort are presented in Table 1. All results presented in the following sections refer to the RWCP cohort with imputation.

Overall, both unadjusted cohorts were comparable. The amivantamab cohort included more patients with an ECOG performance status of one compared to the unadjusted RWPC cohort (71.1% versus 58.2%, respectively). A higher proportion of patients in the amivantamab cohort had pre-treated brain metastases (25.4%) and/or lymph node metastases (54.4%) compared with the RWPC cohort (14.5% and 41.8%, respectively). Liver metastases and other metastatic locations were more frequently observed in the RWPC cohort (20.0% and 70.9%, respectively) compared with the amivantamab cohort (11.4% and 36.8%, respectively).

### 3.2. Treatments Received as Part of RWPC

The treatment classes received as part of RWPC in CATERPILLAR-RWE are described in Table 2. This demonstrates that the treatments previously received by patients were highly heterogenous and used in several combinations. The most commonly named therapy class received in the RWPC cohort was non-platinum-based chemotherapy (27.3%) and the highest percentage of LOTs received ‘Other’ treatments (38.2%); among ‘Other’ treatments, the most common treatments were mobocertinib (ten LOTs) and platinum-based chemotherapy (seven LOTs).

### 3.3. Inverse Probability Weighting

The distribution of PS by the treatment arm before and after ATT adjustment is presented in Figure S1. Overlap between distributions was high before adjustment and was well balanced after ATT adjustment.

The SMDs for each variable before and after IPW-ATT adjustment are shown in Table 1, demonstrating that the characteristics between cohorts became well-balanced following weighting. Following adjustment, SMDs (in the absolute value) were below 0.10 for most (seven) characteristics, and all SMDs were below 0.18, showing that the ATT-weighted RWPC cohort was well balanced versus the amivantamab cohort. Standardized mean differences before and after ATT adjustment are also visually presented in Figure S2.

Following ATT adjustment, both cohorts were comparable. Lymph node metastasis was more frequently observed in the ATT-adjusted RWPC cohort compared with the amivantamab cohort (62.2% versus 54.4%, respectively), whilst adrenal gland metastasis was more frequently observed in the amivantamab cohort versus the ATT-adjusted RWPC cohort (5.3% versus 1.9%, respectively; Table 1). A higher proportion of patients in the amivantamab cohort were age 75 or over compared with the ATT-adjusted RWPC cohort (7.9% and 6.1%, respectively).

### 3.4. Efficacy Assessments

#### 3.4.1. Overall Response Rate

A greater ORR was achieved by the amivantamab cohort compared with the RWCP cohort. The ORR in the amivantamab cohort was 36.8% (*n* = 42) versus the unadjusted ORR of 16.7% (*n* = 9) in the RWPC cohort (Table 3). The ATT-adjusted response rate ratio for amivantamab versus RWPC was 2.14 (1.14, 4.01; *p* = 0.0181). This indicates that patients receiving amivantamab were 2.1 times more likely to achieve a response compared to those who received RWPC.

The response rate ratio estimated for amivantamab versus RWPC from the multivariable analysis (response rate ratio = 2.37 [1.23, 4.58; *p* = 0.0103]) was consistent with the IPW (ATT)-based results.

**Figure 1:**
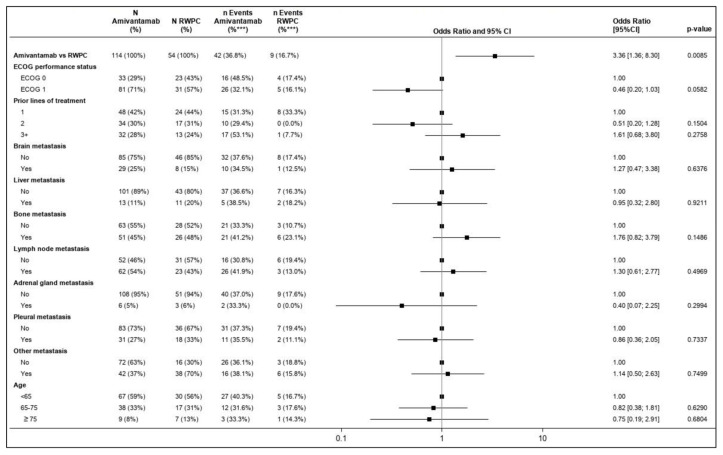
Forest plot of the OR for ORR and CHRYSALIS versus the CATERPILLAR-RWE cohort (amivantamab vs. RWPC)—multivariable regression. *** Percentage was computed as the number of events/N. Percentages presented for ‘N Amivantamab’ and ‘N RWPC’ were rounded. One patient in the RWPC cohort had no information on response and was, therefore, excluded from the analysis of ORR. An OR of the relative treatment effect (first row) greater than 1 favored amivantamab. For each covariate included in the multivariable model, the first level was used as a reference. For the other levels of each covariate, an OR greater than 1 favored improved outcomes relative to the reference category, while accounting for the effect of all other covariates. The square box represents the point estimate, with the horizontal line representing the 95% CIs. CI: confidence interval; ECOG: Eastern Cooperative Oncology Group; HR: hazard ratio; OR: odds ratio; ORR: overall response rate; RWPC: real-world physician’s choice.

The forest plot presented in Figure 1 illustrates the prognostic value for all baseline characteristics for ORR estimated using the multivariable model in terms of OR. As demonstrated in Figure 1, the ECOG’s performance status was identified as potentially prognostic but was not statistically significant (*p* = 0.0582).

#### 3.4.2. Progression Free Survival

**Figure 2:**
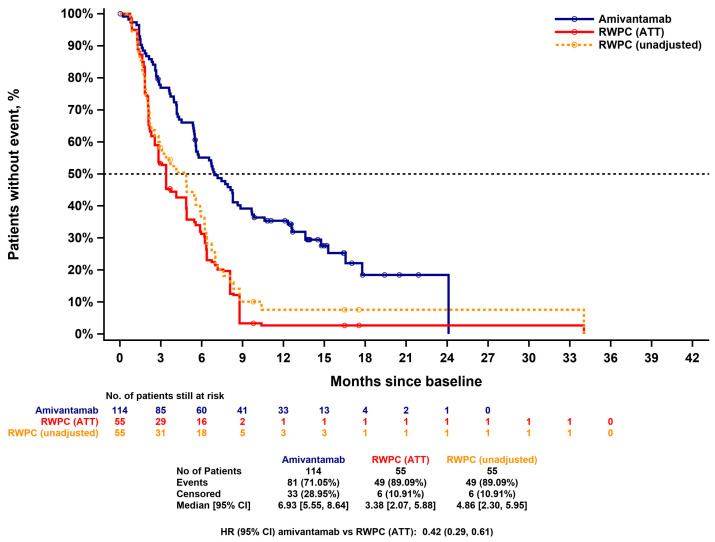
Kaplan–Meier curve for PFS of CHRYSALIS versus CATERPILLAR-RWE (amivantamab versus RWPC)—unadjusted and IPW (ATT)-adjusted. ATT: average treatment effect among the treated; CI: confidence interval; HR: hazard ratio; IPW: inverse probability weighting; PFS: progression-free survival; RWPC: real-world physician’s choice.

The unadjusted and IPW (ATT)-adjusted PFS KM curves for amivantamab and RWPC are presented in Figure 2. The median PFS was 6.93 (5.55, 8.64) months in the amivantamab cohort versus 4.86 (2.30, 5.95) months in the unadjusted RWPC cohort. The ATT-adjusted median of PFS for RWPC was 3.38 months (2.07, 5.88).

The unadjusted HR for the amivantamab cohort versus the RWPC cohort was 0.51 (0.35, 0.74; *p* = 0.0004). The ATT adjusted HR was 0.42 (0.29, 0.61; *p* < 0.0001). The HR estimate for amivantamab versus RWPC from the multivariable analysis was consistent (HR = 0.39; 0.26, 0.58; *p* < 0.0001). This indicates that amivantamab treatment significantly reduced the risk for progression or death (by 58% and 61%, respectively) compared to RWPC.

**Figure 3:**
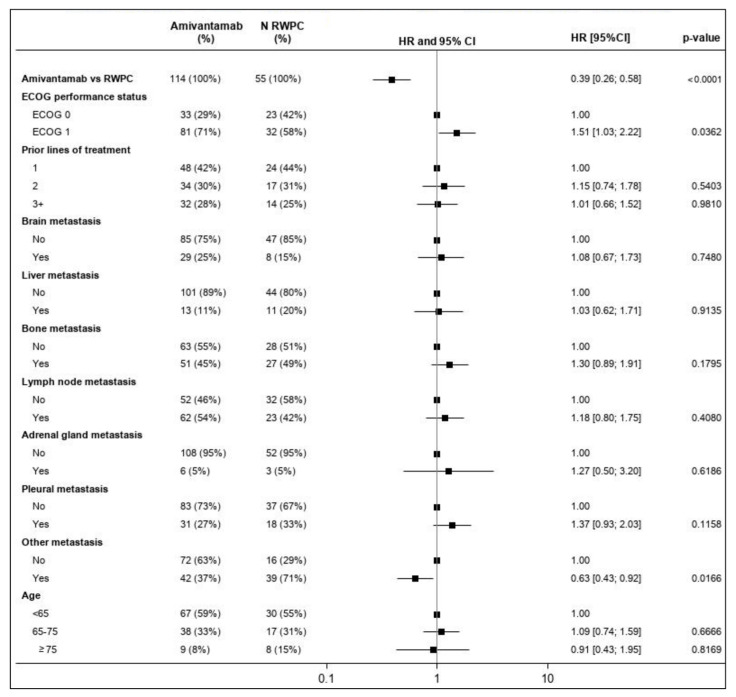
Forest plot of the HR of PFS for CHRYSALIS versus CATERPILLAR-RWE (amivantamab versus RWPC)—multivariable regression. Percentages presented for ‘N Amivantamab’ and ‘N RWPC’ are rounded. An HR of the relative treatment effect (first row) lower than 1 favors amivantamab. For each covariate included in the multivariable model, the first level is used as a reference. For the other levels of each covariate, an HR lower than 1 favors improved outcomes relative to the reference category while accounting for the effect of all other covariates. The square box represents the point estimate, with the horizontal line representing the 95% CIs. CI: confidence interval; ECOG: Eastern Cooperative Oncology Group (performance status); HR: hazard ratio; PFS: progression-free survival; RWPC: real-world physician’s choice.

HR estimates for each baseline characteristic included in the multivariable analysis are summarized in the forest plot in Figure 3. ECOG performance status and other metastatic locations were identified as prognostic.

#### 3.4.3. Time-to-Next-Treatment

**Figure 4:**
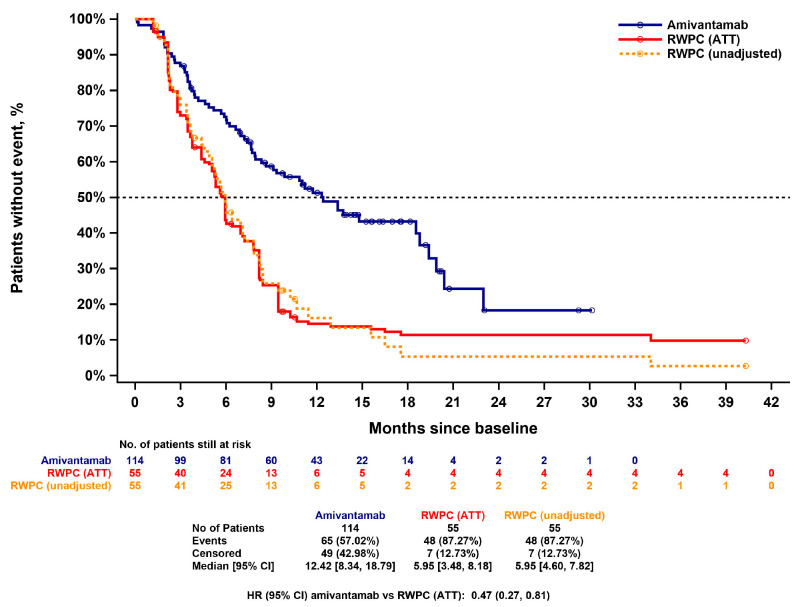
Kaplan–Meier curve of TTNT for CHRYSALIS versus CATERPILLAR-RWE (amivantamab versus RWPC)—unadjusted and IPW (ATT)-adjusted. ATT: average treatment effect among the treated; CI: confidence interval; HR: hazard ratio; IPW: inverse probability weighting; RWPC: real-world physician’s choice; TTNT: time-to-next treatment.

The unadjusted and IPW (ATT)-adjusted TTNT KM curves for amivantamab and RWPC are presented in Figure 4. The median (95% CI) TTNT was 12.42 (8.34, 18.79) months in the amivantamab cohort versus 5.95 (4.60, 7.82) and 5.95 months (3.48, 8.18) in the RWPC cohort prior to and following ATT adjustment, respectively (Figure 4).

The unadjusted HR (95% CI) for the amivantamab cohort versus the RWPC cohort was 0.44 (0.30, 0.64; *p* < 0.0001). ATT-adjusted HR was 0.47 (0.27, 0.81; *p* = 0.0063). The HR estimate for amivantamab versus RWPC from the multivariable analysis was consistent (HR= 0.38; 0.24, 0.61; *p* < 0.0001). This indicates that amivantamab treatment significantly increased TTNT compared to RWPC.

**Figure 5:**
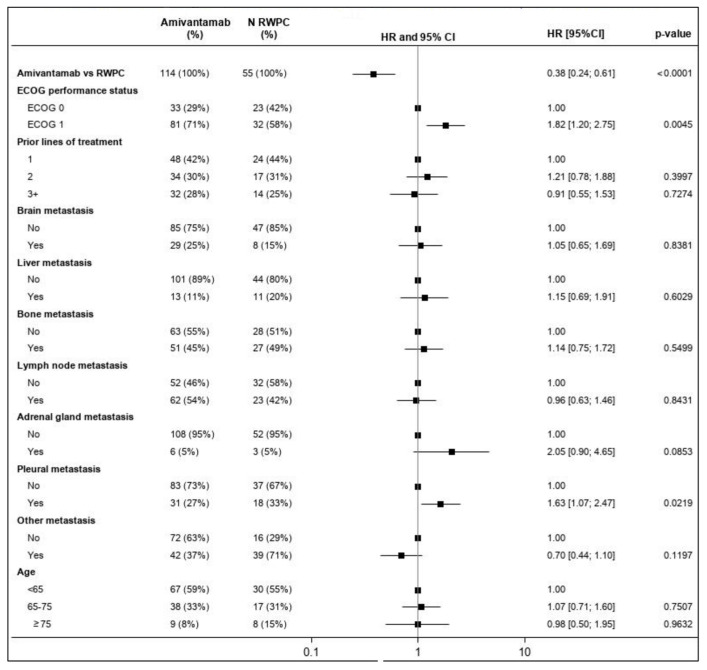
Forest plot of the HR of TTNT for CHRYSALIS versus CATERPILLAR-RWE (amivantamab versus RWPC)—multivariable regression. Percentages presented for ‘N Amivantamab’ and ‘N RWPC’ are rounded. An HR of the relative treatment effect (first row) lower than 1 favors amivantamab. For each covariate included in the multivariable model, the first level is used as a reference. For the other levels of each covariate, an HR lower than 1 favors improved outcomes relative to the reference category while accounting for the effect of all other covariates. The square box represents the point estimate, with the horizontal line representing the 95% CIs. CI: confidence interval; ECOG: Eastern Cooperative Oncology Group (performance status); HR: hazard ratio; RWPC: real-world physician’s choice; TTNT: time-to-next treatment.

HR estimates for each baseline characteristic included in the multivariable analysis are summarized in the forest plot in Figure 5. ECOG performance status and pleural metastases were identified as statistically significant prognostic factors for TTNT; adrenal gland metastases were also identified as a potentially prognostic factor, although this was not statistically significant.

#### 3.4.4. Overall Survival

**Figure 6:**
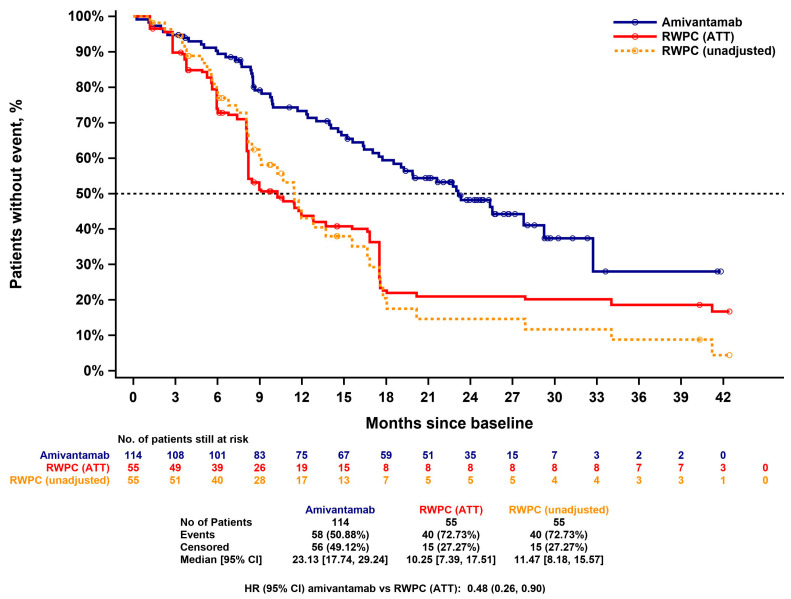
Kaplan–Meier curve of OS for CHRYSALIS versus CATERPILLAR-RWE (amivantamab versus RWPC)—unadjusted and IPW (ATT)-adjusted. ATT: average treatment effect among the treated; CI: confidence interval; HR: hazard ratio; IPW: inverse probability weighting; OS: overall survival; RWPC: real-world physician’s choice.

The unadjusted and IPW (ATT)-adjusted OS KM curves for amivantamab and RWPC are presented in Figure 6. The median OS in the amivantamab cohort was 23.13 months (17.74, 29.24), versus 11.47 months (8.18, 15.57) in the unadjusted RWPC cohort. Following ATT adjustment, the median OS in the RWPC cohort was 10.25 months (7.39, 17.51).

The unadjusted HR for the amivantamab cohort versus the RWPC cohort was 0.42 (0.28; 0.64; *p* < 0.0001). The ATT-adjusted HR was 0.48 (0.26, 0.90; *p* = 0.0207). The HR estimate for amivantamab versus RWPC from the multivariable analysis was consistent (HR = 0.35; 0.21, 0.59; *p* < 0.0001). This indicates that amivantamab treatment significantly reduced the risk for death (by 52% and 65%, respectively) compared to RWPC.

**Figure 7:**
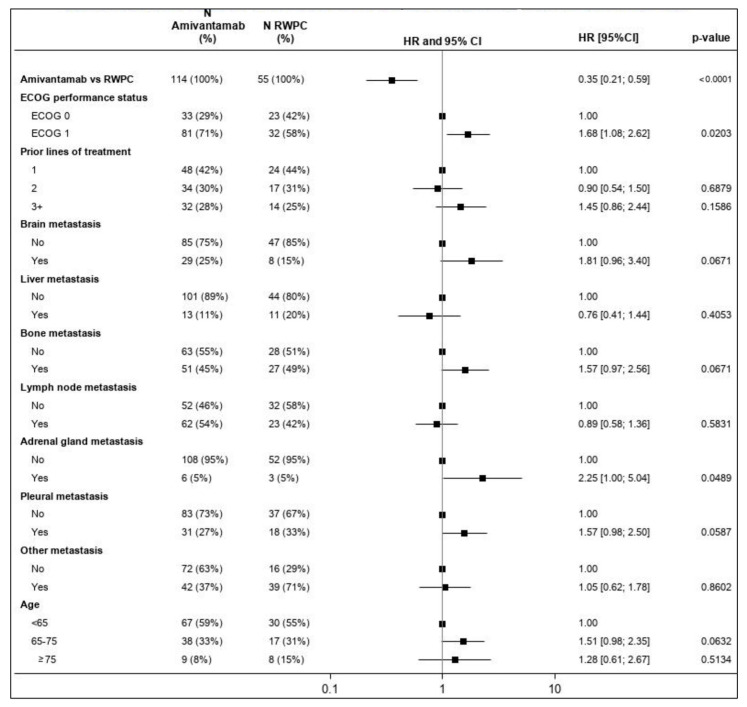
Forest plot of the HR for OS for CHRYSALIS versus CATERPILLAR-RWE (amivantamab versus RWPC)—multivariable regression. Percentages presented for ‘N Amivantamab’ and ‘N RWPC’ are rounded. An HR of the relative treatment effect (first row) lower than 1 favors amivantamab. For each covariate included in the multivariable model, the first level is used as a reference. For the other levels of each covariate, an HR lower than 1 favors improved outcomes relative to the reference category, while accounting for the effect of all other covariates. The square box represents the point estimate, with the horizontal line representing the 95% CIs. CI: confidence interval; ECOG: Eastern Cooperative Oncology Group (performance status); HR: hazard ratio; OS: overall survival; RWPC: real-world physician’s choice.

HR estimates for each baseline characteristic included in the multivariable analysis are summarized in the forest plot in Figure 7. ECOG performance status and adrenal gland metastases were statistically significant prognostic factors for OS; brain, bone, and pleural metastases were identified as potentially prognostic of OS but not statistically significant.

## 4. Discussion

Patients with advanced *EGFR* Exon20ins-mutated NSCLC receiving RWPC treatment face a poor prognosis, as illustrated by the low response rates and survival of patients in the RWPC cohort (ORR of 17.2%, median PFS of 3.38 months and median OS of 10.25 months, following adjustment), which is substantiated by previous studies [14,30]. The RWPC received by these patients is highly heterogenous, as demonstrated by the mix of treatments received in CATERPILLAR-RWE. As such, there is a high unmet need for effective, targeted treatments for advanced *EGFR* Exon20ins-mutated NSCLC.

As highlighted in the introduction, the rarity of *EGFR* Exon20ins mutations, competitive recruitment for clinical studies, and the prior lack of standard of care in this patient population limit the feasibility of a randomized trial in this setting. Therefore, the current analysis represents the best alternative to provide comparative data for the effectiveness of amivantamab versus currently used treatments in Europe across multiple outcomes. This analysis supports the findings of earlier published analyses versus RWPC based on combined US-based real-world sources and national registry data from seven European countries [14,30]. The lack of a standard of care prior to the availability of more targeted treatments for *EGFR* Exon20ins mutations also means that RWPC represents the most relevant comparator to evaluate the relative efficacy of amivantamab as it reflects the heterogeneity of treatments received by this patient population.

In this analysis, a control arm was generated based on data from individual patient records collected in a pan-European chart review study, CATERPILLAR-RWE. The chart review methodology of CATERPILLAR-RWE enabled the collection of richer data compared to those available through national registries alone. In addition, making comparisons against a cohort of patients from diverse sites located in various countries reflects different healthcare systems, representing a robust external comparator. This resembles what was conducted in a randomized clinical study, thereby enriching this comparison.

The adjusted treatment comparisons were conducted using a robust statistical methodology. Unadjusted baseline characteristics were generally comparable between both cohorts and were further balanced after adjustment. Prognostic variables, which were identified as clinically important by an evidence-based process, and which were available in CATERPILLAR-RWE, were adjusted for if the data allowed. Two methods (IPW-ATT and multivariable modelling) were employed to adjust for differences in prognostic baseline characteristics and address potentially related confounding. The results were generally consistent across both methods, indicating the robustness of the analysis.

These analyses demonstrate a clinically and statistically significant treatment benefit for amivantamab versus RWPC across all efficacy outcomes and adjustment methods in a cohort of patients who are representative of a European population with advanced NSCLC with *EGFR* Exon20ins mutations after platinum-based therapy. For amivantamab versus RWPC using IPW-ATT adjustment, patients treated with amivantamab were 2.1 times more likely to achieve an overall response (at least a partial response) and had reduced risk of progression or death and reduced risk of death by 58% and 52%, respectively. The results obtained are consistent with comparative analyses of amivantamab versus a US RWE cohort and analyses of amivantamab versus a pooled EU + US cohort [14].

Safety results were not within the scope of this analysis. Adverse events (AEs) were rigorously captured in CHRYSALIS [17,37]; however, only 45.5% of LOTs in CATERPILLAR-RWE had AEs reported. Therefore, it is likely that AEs were underreported in CATERPILLAR-RWE, limiting the usefulness of a comparison of safety data between these two cohorts.

While comparative analyses were adjusted for a wide range of clinically important prognostic variables, residual confounding could not be entirely excluded. Patients enrolled in clinical trials were considered fit enough for screening and survival until the initiation of treatment, which may potentially result in patients in CHRYSALIS being fitter than those receiving RWPC beyond measured confounders. However, given the strength of the treatment effect across all endpoints, it is highly unlikely that any residual confounding could explain solely the estimated effect [38,39].

Owing to the non-standardized collection of baseline disease characteristics in the real world, certain inclusion criteria from the CHRYSALIS Cohort D+ could not be applied to CATERPILLAR-RWE. In addition, due to the missingness of data or data unavailability, it was not possible to adjust for all baseline characteristics identified as relevant prognostic factors or conduct subgroup analyses based on certain variables. For example, the *EGFR* Exon20ins-specific genotype could not be adjusted for, as there was a high rate of missing values in CATERPILLAR-RWE. In addition, *TP53* mutation status was not available in CHRYSALIS and had a high rate of missing values in CATERPILLAR-RWE. Future studies could additionally analyze the potential effect of *TP53* mutations on the efficacy of amivantamab, as the *TP53* co-mutations have been identified to influence the outcome of NSCLC patients with *EGFR* Exon20ins under RWPC [40].

Non-standardized patient assessments in the real world may result in differences in outcomes between CHRYSALIS and CATERPILLAR-RWE. Therefore, an INV assessment for ORR and PFS from CHRYSALIS was chosen for the primary analysis to align more closely with the measurement of ORR and PFS in real-world clinical practice.

Due to small sample sizes, comparisons of amivantamab versus specific individual treatments or treatment classes were not feasible. An analyses comparing amivantamab to individual treatment classes have been published elsewhere and demonstrate a relative treatment effect that is consistently in favor of amivantamab [13].

## 5. Conclusions

Overall, these analyses provide evidence of clinical and statistical benefits that are in favor of amivantamab versus RWPC, in terms of ORR, PFS, TTNT and OS, in patients with advanced NSCLC and *EGFR* Exon20ins at 2L+. Despite the small sample size of the RWPC cohort, these analyses represent the best alternative to provide comparative data for the effectiveness of amivantamab versus currently used treatments in Europe across multiple outcomes in the absence of data from randomized controlled trials. These results are in line with previously published analyses of amivantamab versus European and US real-world data sources [14].

The poor performance of the RWPC cohort demonstrates the insufficient efficacy of previously applied treatments for this hard-to-treat patient population prior to the release of targeted therapies, such as amivantamab. The comparative efficacy estimates highlight the potential of amivantamab as a highly effective therapy to address this unmet need. Larger randomized studies for amivantamab, such as the phase 3 PAPILLON study comparing the combination of amivantamab and carboplatin-pemetrexed therapy versus carboplatin-pemetrexed in treatment-naïve participants with advanced or metastatic NSCLC with *EGFR* Exon20ins mutations, may help to further validate the benefits of amivantamab in the future [41].

## Figures and Tables

**Table 1:** Baseline characteristics of treatment lines for CHRYSALIS versus the unadjusted and IPW (ATT)-adjusted RWPC cohort.

	Amivantamab	RWPC (Observed)	RWPC (with Imputation)	ATT-Adjusted RWPC	SMD (after ATT Adjustment)
ECOG performance status					
N	114	55	55	55	−0.060
0	33 (28.9%)	23 (41.8%)	23 (41.8%)	14 (26.3%)	
1	81 (71.1%)	32 (58.2%)	32 (58.2%)	41 (73.7%)	
Prior lines of treatment					
N	114	55	55	55	0.024
1	48 (42.1%)	24 (43.6%)	24 (43.6%)	24 (43.2%)	
2	34 (29.8%)	17 (30.9%)	17 (30.9%)	16 (29.4%)	
3+	32 (28.1%)	14 (25.5%)	14 (25.5%)	15 (27.3%)	
Brain metastasis					
N	114	55	55	55	0.068
No	85 (74.6%)	39 (70.9%)	47 (85.5%)	43 (77.5%)	
Yes	29 (25.4%)	7 (12.7%)	8 (14.5%)	12 (22.5%)	
Missing	0	9 (16.4%)	0	0	
Liver metastasis					
N	114	55	55	55	0.061
No	101 (88.6%)	37 (67.3%)	44 (80.0%)	50 (90.5%)	
Yes	13 (11.4%)	9 (16.4%)	11 (20.0%)	5 (9.5%)	
Missing	0	9 (16.4%)	0	0	
Bone metastasis					
N	114	55	55	55	0.058
No	63 (55.3%)	24 (43.6%)	28 (50.9%)	32 (58.1%)	
Yes	51 (44.7%)	22 (40.0%)	27 (49.1%)	23 (41.9%)	
Missing	0	9 (16.4%)	0	0	
Lymph node metastasis					
N	114	55	55	55	−0.159
No	52 (45.6%)	27 (49.1%)	32 (58.2%)	21 (37.8%)	
Yes	62 (54.4%)	19 (34.5%)	23 (41.8%)	34 (62.2%)	
Missing	0	9 (16.4%)	0	0	
Adrenal gland metastasis					
N	114	55	55	55	0.179
No	108 (94.7%)	43 (78.2%)	52 (94.5%)	54 (98.1%)	
Yes	6 (5.3%)	3 (5.5%)	3 (5.5%)	1 (1.9%)	
Missing	0	9 (16.4%)	0	0	
Pleural metastasis					
N	114	55	55	55	0.006
No	83 (72.8%)	31 (56.4%)	37 (67.3%)	40 (73.1%)	
Yes	31 (27.2%)	15 (27.3%)	18 (32.7%)	15 (26.9%)	
Missing	0	9 (16.4%)	0	0	
Other metastasis					
N	114	55	55	55	0.043
No	72 (63.2%)	10 (18.2%)	16 (29.1%)	36 (65.2%)	
Yes	42 (36.8%)	36 (65.5%)	39 (70.9%)	19 (34.8%)	
Missing	0	9 (16.4%)	0	0	
Age					
N	114	55	55	55	0.136
<65	67 (58.8%)	30 (54.5%)	30 (54.5%)	30 (54.9%)	
65–75	38 (33.3%)	17 (30.9%)	17 (30.9%)	21 (39.1%)	
≥75	9 (7.9%)	8 (14.5%)	8 (14.5%)	3 (6.1%)	

Percentages for the IPW ATT-adjusted cohort cannot be replicated due to rounding in the reported frequency count. SMD is based on RWPC after the imputation of missing values. ATT: average treatment effect among the treated; ECOG: Eastern Cooperative Oncology Group (performance status); IPW: inverse probability weighting; RWPC: real-world physician’s choice; SMD: standardized mean difference.

**Table 2:** Treatments received as part of RWPC in CATERPILLAR-RWE.

Treatment Class	Number of LOTs (%)
IO	7 (12.7)
EGFR TKI	9 (16.4)
Non-platinum-based chemotherapy	15 (27.3)
VEGFi + chemotherapy	3 (5.5)
Other ^1^	21 (38.2)

Percentages do not sum to 100% due to rounding. ^1^ ‘Other’ consisted of atezolizumab, bevacizumab, carboplatin and paclitaxel (*n* = 1), carboplatin and gemcitabine (*n* = 1), carboplatin and paclitaxel albumin (*n* = 2), carboplatin and pemetrexed (*n* = 4), cetuximab (*n* = 1), crizotinib (*n* = 1), mobocertinib (*n* = 10), and poziotinib (*n* = 1). Even though atezolizumab, bevacizumab, carboplatin and paclitaxel contain platinum-based chemotherapy (carboplatin), this is not the primary part of the regimen and, therefore, this is not considered a platinum-based chemotherapy regimen. EGFR: epidermal growth factor receptor; IO: immuno-oncology agent; LOT: line of therapy; RWPC: real-world physician’s choice; TKI: tyrosine kinase inhibitor; VEGFi: vascular endothelial growth factor inhibitor.

**Table 3:** Unadjusted and adjusted ORR for the CHRYSALIS versus RWPC cohort (amivantamab versus RWPC).

Method	ORR		OR (95% CI)	*p* Value	Response Rate Ratio (95% CI)	*p* Value
	Amivantamab	RWPC				
Unadjusted comparison	36.8%	16.7%	2.92 (1.30, 6.56)	0.0096	2.21 (1.16, 4.20)	0.0156
IPW–ATT approach	36.8%	17.2%	2.80 (1.26, 6.22)	0.0116	2.14 (1.14, 4.01)	0.0181
Multivariable regression	29.7%	11.1%	3.36 (1.36, 8.30)	0.0085	2.37 (1.23, 4.58)	0.0103

One patient in the RWPC cohort had no information on response and was, therefore, excluded from the analysis of ORR. ATT: average treatment effect among the treated; CI: confidence interval; IPW: inverse probability weighting; OR: odds ratio; ORR: overall response rate; RWPC: real-world physician’s choice.

## Data Availability

The materials, data and protocols associated with the current analysis are available from the corresponding author on reasonable request. The data sharing policy of Janssen Pharmaceutical Companies of Johnson & Johnson is available at https://www.janssen.com/clinical-trials/transparency, accessed on 5 September 2023. As noted on this site, requests for access to CHRYSALIS study data can be submitted through the Yale Open Data Access (YODA) Project site at http://yoda.yale.edu, accessed on 5 September 2023.

## Supplementary Materials

The following supporting information can be downloaded at: https://www.mdpi.com/article/10.3390/cancers15225326/s1, Figure S1: Distribution of PS by treatment arm, before and after ATT adjustment of the RWPC cohort; Figure S2: SMD before and after ATT adjustment (RWPC cohort versus amivantamab-treated cohort); Table S1: Key inclusion and exclusion criteria applied to CHRYSALIS Cohort D+; Table S2: Inclusion and exclusion criteria applied to CATERPILLAR-RWE; Table S3: Eligibility criteria of Cohort D+ of the CHRYSALIS trial that could not be applied to the RWPC cohort; Table S4: Baseline characteristics used as covariates in the PS and multivariable regression models; Table S5: Baseline variables not included in the adjustment; Table S6: Selection of treatment lines included in CATERPILLAR-RWE.
