# Supplementary material for: Amivantamab Compared with Real-World Physician’s Choice after Platinum-Based Therapy from a Pan-European Chart Review of Patients with Lung Cancer and Activating EGFR Exon 20 Insertion Mutations
Source: Cancers (Basel). 2023 Nov 8;15(22):5326. doi: 10.3390/cancers15225326 (PMC10670157; doi:10.3390/cancers15225326)
Supplement: Supplementary file 1 [file cancers-15-05326-s001.zip › Supplementary Figures.docx]

Supplementary Material: Figures


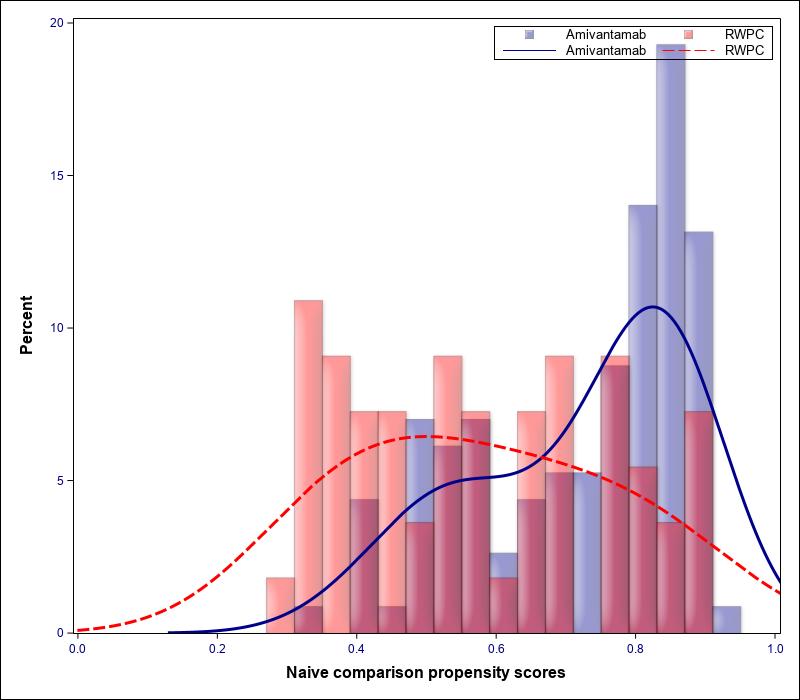

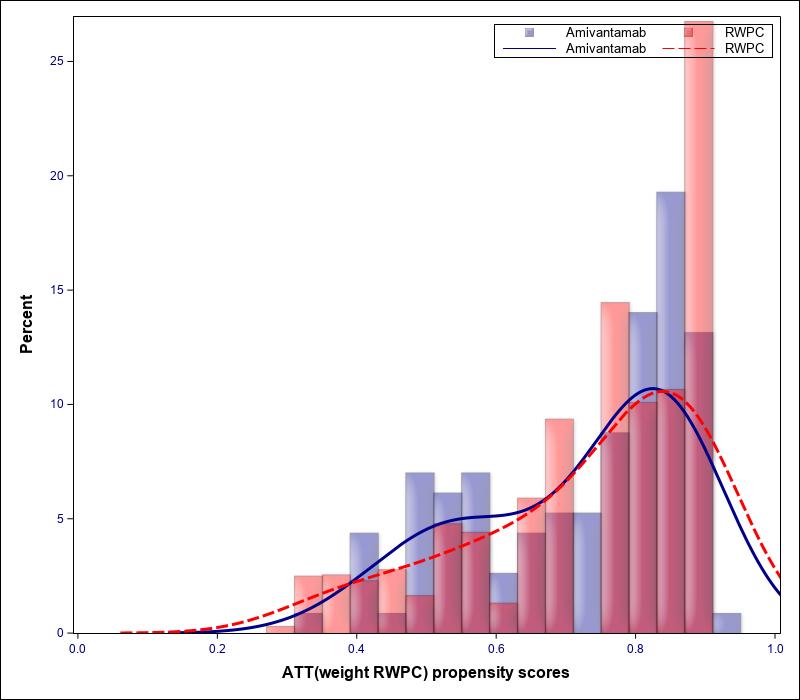


**Figure S1:** Distribution of PS by treatment arm, before and after ATT adjustment of the RWPC cohort.

ATT: average treatment effect among the treated; PS: propensity score; RWPC: real-world physician’s choice.


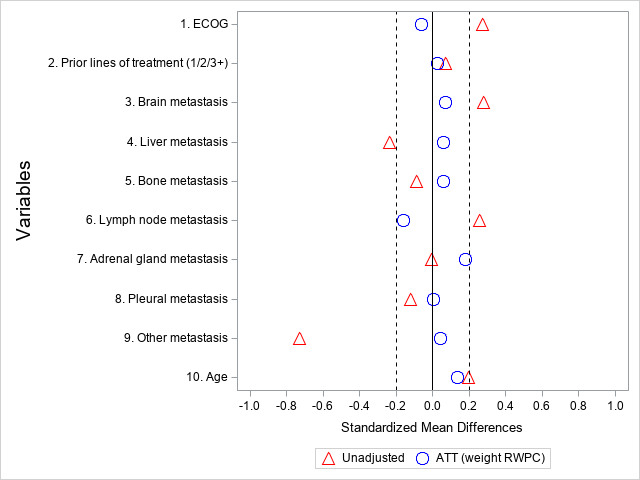


**Figure S2**: SMD before and after ATT adjustment (RWPC cohort versus amivantamab-treated cohort).

SMD is based on RWPC after imputation of missing values. ATT: average treatment effect among the treated; ECOG: Eastern Cooperative Oncology Group (performance status); RWPC: real-world physician’s choice; SMD: standardized mean difference.
