# Supplementary material for: Amivantamab Compared with Real-World Physician’s Choice after Platinum-Based Therapy from a Pan-European Chart Review of Patients with Lung Cancer and Activating EGFR Exon 20 Insertion Mutations
Source: Cancers (Basel). 2023 Nov 8;15(22):5326. doi: 10.3390/cancers15225326 (PMC10670157; doi:10.3390/cancers15225326)
Supplement: Supplementary file 1 [file cancers-15-05326-s001.zip › Supplementary Tables.docx]

Supplementary Material: Tables

**Table S1:** Key inclusion and exclusion criteria applied to CHRYSALIS Cohort D+.

| **Inclusion criteria** | **Exclusion criteria** |
| --- | --- |
| - Stage IIIB/C or IV NSCLC - *EGFR* Exon20ins - Aged ≥18 years - Failure of a platinum-based therapy at any point after initial NSCLC diagnosis - ECOG performance status 0 or 1 - Hemoglobin ≥10 g/dL, ANC ≥1.5 x10^9^/L, platelets ≥75 x10^9^/L, AST and ALT ≤3 x ULN, total bilirubin ≤1.5 ULN (patients with Gilbert’s syndrome can enroll if conjugated bilirubin is within normal limits), serum creatinine <1.5 x ULN or calculated/measured creatinine clearance >50 mL/min/1.73m^2 a^ - No transfusions or use of G-CSF within seven days prior to testing ^a,b^ | - History of significant cardiovascular disease, including, but not limited to, deep vein thrombosis or pulmonary embolism within four weeks prior to the first dose of study drug, myocardial infarction, unstable angina, stroke, transient ischemic attack, coronary/peripheral artery bypass graft or any acute coronary syndrome within six months prior to the first dose of study drug, prolonged QTcF interval, uncontrolled hypertension and congestive heart failure - Untreated brain metastases (patients with definitively, locally-treated metastases that are clinically stable and asymptomatic for at least two weeks and who are off or receiving low-dose corticosteroid treatment [≤10 mg prednisone or equivalent] for at least two weeks prior to study treatment were eligible) - Other primary cancer diagnoses (exceptions include squamous and basal cell carcinomas of the skin and carcinoma in situ of the cervix) within three years prior to screening - Leptomeningeal disease ^a^ - Positive HBsAg or HCV antibody, or other clinically active infectious liver disease - History of HIV antibody positive, or positive for HIV at screening ^a^ - Medical history of ILD, including drug induced ILD or radiation pneumonitis requiring treatment with prolonged steroids or other immune suppressive agents within the last two years ^a^ |

^a^ These eligibility criteria were not applied to CATERPILLAR-RWE. ^b^ Testing refers to the blood tests described in previous bullet points. ALT: alanine aminotransferase; ANC: absolute neutrophil count; AST: aspartate aminotransferase; ECOG: Eastern Cooperative Oncology Group (performance status); *EGFR*: epidermal growth factor receptor; Exon20ins: Exon 20 insertion; G-CSF: granulocyte colony stimulating factors; HBsAg: hepatitis B surface antigen; HCV: hepatitis C virus; HIV: human immunodeficiency virus; ILD: interstitial lung disease; NSCLC: non-small cell lung cancer; ULN: upper limit of normal.

**Table S2:** Inclusion and exclusion criteria applied to CATERPILLAR-RWE.

| **Inclusion criteria** | **Exclusion criteria** |
| --- | --- |
| - Stage IIIB/C or IV NSCLC diagnosed between 01 January 2011 and 31 July 2020 - *EGFR* Exon20ins ^a^ - Aged ≥18 years - Received at least one subsequent LOT following the date of obtention of the tissue (e.g. biopsy, surgical piece) on which the EGFR Exon20ins mutation was identified - Received at least one subsequent LOT following progression on/after a platinum-based SACT in the advanced setting - Prior treatment line with platinum-based SACT was administered: following the diagnosis of metastatic NSCLC, or for locally advanced disease, given in the 12 months prior to diagnosis of metastatic disease - Provision of signed informed consent form for data collection, as applicable based on country specific requirements (exceptions: waiver for patients who are alive, lost to follow-up or deceased [based on site specific regulation]) | - A line of treatment with a record of ECOG performance status > 1, or Karnofsky index ≤ 90% if ECOG is not available, within 60 days prior to the start of the LOT - Cardiovascular comorbidity at the date of initiation of the subsequent LOT following progression on/after a platinum-based SACT (uncontrolled hypertension, congestive heart failure, pericarditis, myocarditis) or prior to this date (within six months: myocardial infarction, unstable angina, stroke, transient ischemic attack, coronary/peripheral artery bypass graft, or any acute coronary syndrome) - Other malignancy within three years prior to diagnosis of locally advanced or metastatic NSCLC, with the exception of non-melanoma skin cancer - Clinically active infectious liver disease at the date of initiation of the subsequent LOT following progression on/after a platinum-based SACT - Untreated brain metastases (patients with locally-treated brain metastases that were clinically stable and asymptomatic were eligible) |

^a^ Previously diagnosed on fresh or archival tissue (as identified in the patient medical records). No biopsy or genomic analysis was performed for the study. *EGFR*: epidermal growth factor receptor; ECOG: Eastern Cooperative Oncology Group; Exon20ins: Exon 20 insertion; LOT: line of therapy; NSCLC: non-small cell lung cancer; SACT: systemic anti-cancer therapy.

**Table S3:** Eligibility criteria of Cohort D+ of the CHRYSALIS trial that could not be applied to the RWPC cohort.

| **Inclusion criteria** | **Exclusion criteria** |
| --- | --- |
| - Hemoglobin ≥10 g/dL - ANC ≥1.5 x10^9^/L - Platelets ≥75 x10^9^/L - AST and ALT ≤3 x ULN - Total bilirubin ≤1.5 ULN (patients with Gilbert’s syndrome could enroll if conjugated bilirubin was within normal limits) - Serum creatinine <1.5 x ULN or calculated/measured creatinine clearance >50 mL/min/1.73m^2^ - No transfusions or use of G-CSF within seven days prior to testing ^a^ | - Leptomeningeal disease - Medical history of ILD, including drug induced ILD or radiation pneumonitis requiring treatment with prolonged steroids or other immune suppressive agents within the last two years |

^a^ Testing refers to the blood tests described in previous bullet points. ALT: alanine aminotransferase; ANC: absolute neutrophil count; AST: aspartate aminotransferase; G-CSF: granulocyte colony stimulating factors; ILD: interstitial lung disease; RWPC: real-world physician’s choice; ULN: upper limit of normal.

**Table S4:** Baseline characteristics used as covariates in the PS and multivariable regression models.

| **Variable** | **Categories** |
| --- | --- |
| 1: ECOG performance status | 0, 1 |
| 2: Number of previous lines of treatment | 1, 2, 3+ |
| 3: Bone metastases | Yes, No |
| 4: Liver metastases | Yes, No |
| 5: Brain metastases | Yes, No |
| 6: Lymph node metastases | Yes, No |
| 7: Adrenal gland metastases | Yes, No |
| 8: Other metastatic locations: lung, peritoneum, nervous system, pleura or other localization different than the previous ones | Yes, No |
| 9: Age category | <65, 65 to <75, ≥75 |

Baseline characteristics are measured at initiation of each RWPC LOT. ECOG: Eastern Cooperative Oncology Group; PS: propensity score.

**Table S5:** Baseline variables not included in the adjustment.

| **Variable** | **Main reason for exclusion** |
| --- | --- |
| Ethnicity | High missingness in CATERPILLAR-RWE |
| Body mass index | Inclusion did not achieve balance among covariates |
| *EGFR* Exon20ins variant | High missingness in CATERPILLAR-RWE |
| *TP53* mutation | Not available in CHRYSALIS and high missingness in CATERPILLAR-RWE |
| Smoking history | Inclusion did not achieve balance among covariates |
| NSCLC stage at initial diagnosis | Inclusion did not achieve balance among covariates |
| Any surgery or procedure for NSCLC | High missingness in CATERPILLAR-RWE |
| Sex | Inclusion did not achieve balance among covariates |
| Re-biopsy | High missingness in CATERPILLAR-RWE |
| Tumor PD-L1 status | Not available in CHRYSALIS and high missingness in CATERPILLAR-RWE |
| Liver function abnormality | High missingness in CATERPILLAR-RWE |

*EGFR*: epidermal growth factor receptor; Exon20ins: Exon 20 insertion; NSCLC: non-small cell lung cancer; PD-L1: programmed death-ligand 1.

**Table S6:** Selection of RWCP treatment lines included in CATERPILLAR-RWE.

|  | **RWPC** |
| --- | --- |
| Patients included in the study | 93 |
| Screen failures | 10 |
| LOTs of patients who were not screen failures | 277 LOTs |
| Of which, post-platinum | 188 LOTs |
| Of which, ECOG performance status <2 (if not missing) | 170 LOTs |
| Of which, excluding untreated brain metastases | 160 LOTs |
| Of which, Exon20ins diagnosis before start of LOT | 134 LOTs |
| Of which, excluding amivantamab or post-amivantamab | 130 LOTs |
| Of which, non-missing ECOG performance status | 55 LOTs |
| Unique patients in RWPC | 38 |

Listed criteria by LOT are applied sequentially. Criteria not listed in this table were satisfied for all LOTs included in the final RWPC cohort. ECOG: Eastern Cooperative Oncology Group; Exon20ins: Exon 20 insertion; LOT: line of treatment; RWPC: real-world physician’s choice.
